# Machine Learning–Integrated Metabolomics for Precision Pharmacotherapy: Advances, Challenges, and Clinical Translation

**DOI:** 10.3390/metabo16080600

**Published:** 2026-08-21

**Authors:** Pan Li, Jing Mao, Xianglin Hu, Yujiao Hu, Xiaoke Zhang, Qian Zheng, Xiaoying Hou, Yuchen Liu, Min Huang

**Affiliations:** 1 Cancer Institute, School of Medicine, Jianghan University, Wuhan 430056, China; panli@jhun.edu.cn (P.L.);; 2 Institute of Clinical Pharmacology, School of Pharmaceutical Sciences, Sun Yat-sen University, Guangzhou 510006, China; 3 Department of Pharmacy, Shenzhen Children’s Hospital, Shenzhen 518026, China; 4 Hubei Key Laboratory of Cognitive and Affective Disorders, Jianghan University, Wuhan 430056, China; 5 Hubei Provincial Demonstration Center for Experimental Medicine Education, School of Medicine, Jianghan University, Wuhan 430056, China

**Keywords:** machine learning, metabolomics, precision pharmacotherapy, drug response prediction, adverse drug reactions

## Abstract

Machine learning (ML) integrated with metabolomics has emerged as a promising strategy to advance precision pharmacotherapy, enabling data-driven prediction of drug response. This review provides an overview of commonly applied ML methodologies in metabolomics-based pharmacological studies, including supervised models (Random Forest, Extreme Gradient Boosting, Support Vector Machine, Logistic Regression, K-Nearest Neighbors), unsupervised models (K-Means Clustering, Principal Component Analysis), and deep learning approaches. We summarize recent progress in the application of metabolomics-driven ML to personalized medication, with a focus on drug dosage optimization, therapeutic efficacy prediction, and adverse drug reaction assessment. Despite these advances, significant challenges remain, including limited explainability, insufficient prospective clinical validation, lack of standardization and reproducibility, and data dimensionality and quality issues. Addressing these issues will be essential for the clinical translation of ML-metabolomics integration. Looking ahead, continued methodological innovation, large-scale multi-center prospective validation, and integration with other omics platforms will be key to unlocking the full potential of metabolomics combined with ML in precision healthcare.

## 1. Introduction

The core principle of clinical drug administration is to give the right drug, at the right dose, by the right route, at the right time, to the right patient. However, interindividual variability in drug response remains a major challenge in clinical pharmacotherapy, often leading to suboptimal efficacy or severe adverse drug reactions [1]. Pharmacogenomics and pharmacokinetic studies have improved the understanding of drug response variability and have been integrated into clinical practice for certain drugs. Pharmacogenomics has catalyzed a paradigm shift in healthcare, from the conventional “one-size-fits-all” model toward precision, patient-specific therapies [2]. However, these approaches explain only a part of the observed interindividual variability.

Metabolomics, which provides an integrated view of both genetic and environmental influences, has emerged as a powerful tool in precision pharmacotherapy [3]. Supported by highly sensitive detection technologies, metabolomics enables comprehensive profiling of endogenous metabolites in biological samples. Unlike genomics and transcriptomics, which reflect genetic potential and gene expression, metabolomics directly captures the dynamic metabolic shifts induced by disease progression and pharmacological interventions. In pharmacokinetic studies, metabolomics can use untargeted or targeted analyses and be combined with other omics technologies to track metabolic pathways associated with drug clearance [4]. At the same time, databases such as HMDB [5] and MetaboAnalyst [6] provide a vital platform for analyzing drug metabolism mechanisms. These advantages enable metabolomics to offer key insights for optimizing treatment strategies.

However, metabolomics data is characterized by high dimensionality, strong nonlinearity, and large individual differences, making it difficult for traditional statistical methods to extract reliable predictive patterns. ML algorithms have emerged as a powerful solution for this challenge [7]. They excel at extracting key variables and constructing personalized models from high-dimensional, nonlinear data. Combined with Explainable AI (XAI) such as SHapley Additive exPlanations (SHAP) and Local Interpretable Model-agnostic Explanations (LIME), it can enhance the interpretability of model predictions, providing decision support that can be directly adopted in clinical practice [8]. When these two approaches are combined, a new model shifting from post hoc adjustment to pre-treatment prediction is emerging. For example, McCune et al. [9] applied the LASSO model, combined with metabolomics data, screening 13 key metabolites out of 841 metabolites, and successfully constructed a predictive model for busulfan clearance. This model explained 40% of the variability in clearance and completed the prediction two weeks before dosing. It not only achieved effective dimensionality reduction in high-dimensional data but also improved predictive accuracy, providing a new pathway for precision pharmacotherapy.

This review focuses on ML-driven metabolomics in precision pharmacotherapy. We first introduce mainstream algorithms and their applicable scenarios and then summarize the current state of research in three key areas, including drug dose optimization, efficacy prediction, and adverse drug reaction prediction. Finally, we discuss the challenges and prospects of clinical application, intending to provide novel insights into the effective and ethical deployment in precision pharmacotherapy.

## 2. Machine Learning Concepts Relevant to Metabolomics

### 2.1. Supervised Learning

Supervised learning is a paradigm in which a model learns a function from a set of labeled training samples, where each input data is paired with a known output. This process enables the estimation of model parameters and the construction of an optimal predictive model from data [10]. Common supervised learning algorithms include ANN, RF, XGBoost, SVM, Logistic Regression, and KNN, among others.

#### 2.1.1. Random Forest

**Figure 1:**
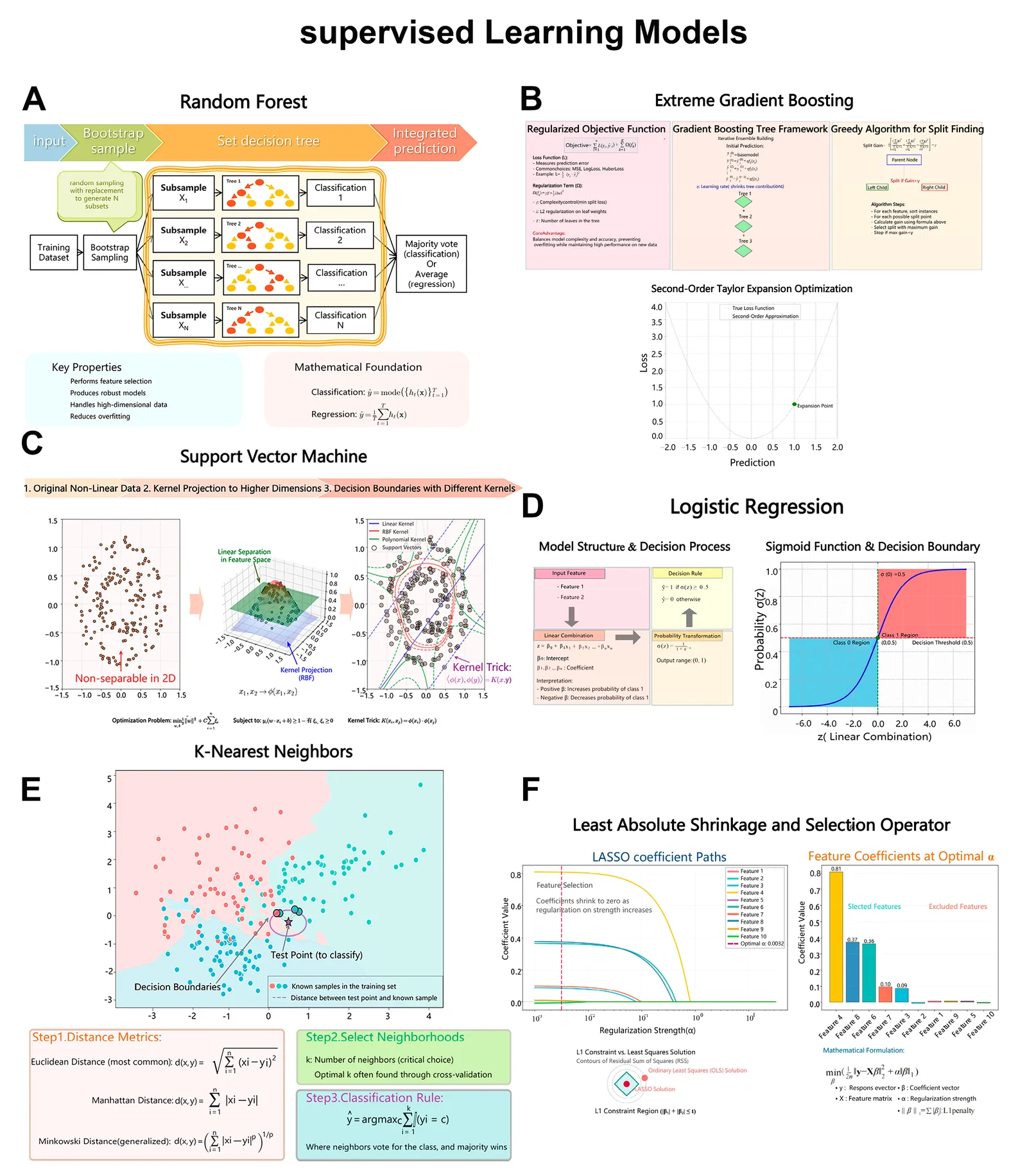
Schematic illustration of supervised machine learning algorithms used in metabolomics analysis. These models are trained on labeled metabolomic datasets to classify samples or predict clinical outcomes. Ensemble methods such as Random Forest (**A**) and XGBoost (**B**) enhance prediction accuracy through multiple weak learners; SVM (**C**) constructs optimal separating hyperplanes in high-dimensional space; Logistic Regression (**D**) estimates outcome probabilities; KNN (**E**) classifies samples based on feature proximity, it estimates outcome probabilities based on feature proximity, with red and blue regions indicating class-specific prediction areas and the boundary representing the decision threshold and LASSO (**F**) performs variable selection by penalizing regression coefficients, thereby improving model interpretability and reducing overfitting.

Random Forest (RF) is an ensemble learning model based on the bagging framework that incorporates feature randomization, in which multiple decision trees (base learners) are trained in parallel and their outputs aggregated via majority voting or averaging (Figure 1A) [11]. RF is valued for its strong predictive accuracy and stability when applied to small-sample and high-dimensional datasets [12]. Bagging and classification and regression tree (CART) splitting schemes play pivotal roles in the RF mechanism; rigorous mathematical analysis of either component remains challenging, which explains why simplified versions of the original procedure have been considered in theoretical research to date. RF is a further improvement of the CART model. The decision tree algorithm operates through recursive partitioning, which selects optimal decision thresholds for variables by evaluating multiple candidate thresholds per predictor and implementing the most discriminative threshold until further splits cease to improve discriminatory power [13]. Leveraging intrinsic feature selection and high resilience to noise, RF has been widely applied in precision medicine for tasks including adverse drug reaction early-warning systems, anticancer drug sensitivity prediction, personalized treatment allocation, identification of high-risk medication factors, and interpretable decision support.

#### 2.1.2. Extreme Gradient Boosting

Extreme Gradient Boosting (XGBoost) is an efficient algorithm. The core advantage stems from its unique regularized learning mechanism and the deep integration of the gradient boosting tree framework (Figure 1B). By constructing the regularized objective function, the algorithm can combine the loss function with the regularization penalty term, thus ensuring that the model still maintains high accuracy in the face of new data. In the model training mechanism, XGBoost adopts an iterative enhancement strategy to build a gradient boosting tree integration model. In each iteration, the algorithm generates a new decision tree to correct the residual errors of the preceding model by introducing a second-order Taylor expansion to optimize the loss function and employing a greedy algorithm to search for the best split point [14]. This structured tree-growing method enables the model to automatically capture non-linear relationships and higher-order interaction effects. Therefore, XGBoost can intelligently identify key predictors such as key biomarkers, medication histories, and genetic features from a large number of medical records and reveal potential risk factors through feature importance ranking, making it particularly suitable for adverse reaction and drug efficacy prediction in precision pharmacotherapy [15].

#### 2.1.3. Support Vector Machine

Support Vector Machine (SVM) is a supervised learning algorithm that performs classification or regression by constructing an optimal hyperplane (or set of hyperplanes) that maximizes the geometric margin between classes (Figure 1C) [16]. For nonlinearly separable data, the kernel trick is employed to map the input space into a high-dimensional feature space where linear separation becomes feasible. SVM mechanisms include linear classification, nonlinear kernel-based transformations, and parameter optimization. Compared with other methods, logistic regression is a probabilistic model well-suited for linearly separable problems but cannot inherently handle nonlinear patterns; decision trees offer high interpretability but are sensitive to high-dimensional sparse data; neural networks can capture complex patterns but require large datasets and significant computational resources. In contrast, SVM demonstrates stability in high-dimensional spaces and retains an advantage in small-sample scenarios due to its solid theoretical foundations and strong generalization capability. Subsequent improved models, such as Support Vector Regression (SVR) and accelerated versions based on approximation algorithms (e.g., LIBSVM), have further expanded the scope of application [17].

#### 2.1.4. Logistic Regression

Logistic Regression is a classification model constructed based on the idea of linear regression, which is often used to study the influence of multiple factors on dichotomous classification results, and its core principle is to convert the contribution of the independent variable into a probability value through the logit function (Figure 1D) [18]. Firstly, the weighted summation of the variables obtains a composite score, and then the score is transformed into a probability between 0 and 1. Finally, the classification result is judged according to the probability threshold. This transformation process retains the interpretability of linear models while enabling them to handle nonlinear relationships in classification problems. This makes it particularly well-suited for precision pharmacotherapy. When working with limited sample sizes, it allows researchers to prioritize clinically significant variables and enhance predictive accuracy while controlling the model complexity [19].

#### 2.1.5. K-Nearest Neighbors

K-Nearest Neighbors (KNN) is a classical supervised learning algorithm based on similarity metrics. Its core idea follows the intuitive logic of “clustering things into categories”. Mechanistically, the model operates by calculating the feature distances between the target sample (such as patient physiological data) and all the samples in the training set (Figure 1E). The algorithm then identifies the K closest neighbors and infers the target label by applying majority voting in classification tasks or averaging in regression tasks [20,21]. KNN demonstrates unique value in the field of precision pharmacotherapy with its strengths in handling non-linear relationships and complex biological data [21]. The core feature that distinguishes KNN from other ML models is that it only relies on the local distribution characteristics of different data, without explicitly training the model parameters, and performs real-time distance calculations and decision-making [22]. The dynamic adaptability of KNN allows new patient data to be added to the training set directly without re-training the model. Moreover, KNN is inherently small-sample friendly, which helps mitigate the risk of overfitting that often arises in parametric models when applied to limited datasets. This property renders KNN particularly suitable for treatment optimization in rare diseases, where patient data are inherently scarce and personalized medical strategies are essential.

#### 2.1.6. Least Absolute Shrinkage and Selection Operator (LASSO)

The Least Absolute Shrinkage and Selection Operator (LASSO) is a regularization algorithm that is often used to select the variables that are most strongly associated with the outcome of interest (Figure 1F) [23]. Compared with stepwise regression, LASSO offers simultaneous variable selection and optimization of predictor numbers. By imposing an L1 penalty on the absolute values of the regression coefficients, LASSO performs both coefficient shrinkage and variable selection simultaneously [24]. This approach forces some coefficients to be exactly zero, effectively excluding irrelevant or redundant predictors from the model [25]. Compared with traditional regression methods, LASSO reduces model complexity, mitigates multicollinearity, and improves generalizability, particularly in high-dimensional datasets. In biomedical research and precision medicine, LASSO is widely employed to identify key biomarkers, optimize predictive features, and construct robust prognostic models, thereby supporting precision pharmacotherapy decisions and drug response predictions [24,25,26].

### 2.2. Unsupervised Learning

Unsupervised learning operates on unlabeled datasets, where no predefined outputs are available, and the categories or groupings of samples are unknown. The objective is to identify and extract inherent structural patterns within the data. This learning paradigm is widely applied to tasks such as clustering and dimensionality reduction. Representative algorithms include K-Means Clustering and Hierarchical Clustering, as well as dimensionality reduction techniques such as Principal Component Analysis (PCA), among others [27]. Overall, unsupervised learning offers indispensable tools in precision medicine, enabling the extraction of meaningful patterns from unlabeled data and supporting the development of personalized medical strategies.

#### 2.2.1. K-Means Clustering

**Figure 2:**
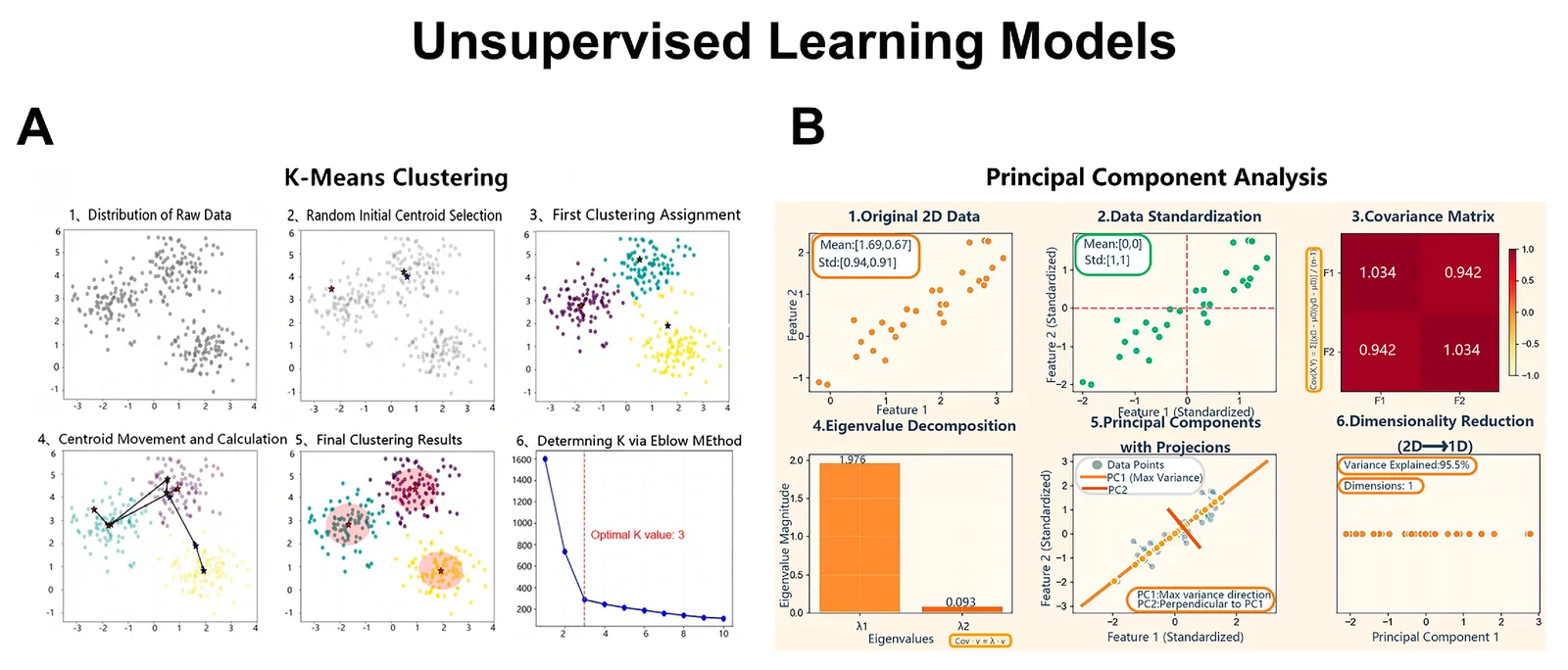
Schematic illustration of unsupervised machine learning algorithms used in metabolomic analysis. (**A**) KMC partitions samples into k distinct groups based on similarity in their metabolite profiles, enabling the identification of intrinsic metabolic subgroups without prior class labels. (**B**) PCA reduces the dimensionality of complex metabolomic datasets by projecting correlated variables onto a smaller number of orthogonal principal components, thereby revealing the major sources of variance in the data. For (**A**), differently colored dots denote samples assigned to distinct K-means clusters. For (**B**), colored dots represent data samples at different processing stages; solid and dashed orange lines indicate the first and second orthogonal principal components, respectively; the color gradient in the heatmap corresponds to the magnitude of covariance values.

K-Means Clustering (KMC) is an unsupervised learning algorithm that divides a dataset into K clusters. Data points within the same cluster have high similarity. This method requires specifying the initial number of K groups in advance. Usually, the average value of all data points within a cluster is taken as the center of that cluster, and an iterative algorithm is used to minimize the distance between each observation and its corresponding average value (Figure 2A). KMC is mainly used for patient stratification and profiling. Miroslava Nedyalkova proposed KMC to accurately classify patients with type 2 diabetes mellitus diagnosed with an underlying disease [28]. KMC relies on calculating the distance between cluster centers and each sample, which is associated with drawbacks such as high computational cost and suboptimal performance [29]. However, KMC has notable limitations, such as susceptibility to local optima when initial cluster centers are poorly chosen. To overcome this, Tang et al. developed the d-k-means algorithm, which incorporates density–distance weighting and applies the min–max principle to automatically determine both the initial centers and their number [30].

#### 2.2.2. Principal Component Analysis (PCA)

Principal Component Analysis (PCA) is one of the most widely used unsupervised dimensionality reduction techniques in biomedical research [31,32]. It transforms high-dimensional data into a lower-dimensional space by identifying orthogonal principal components that capture the maximum variance in the dataset [33]. Each principal component is a linear combination of the original features, ordered by the amount of variance explained, thereby allowing researchers to retain the most informative aspects of the data while reducing noise and redundancy (Figure 2B). In precision medicine, PCA is commonly employed in the analysis of high-throughput omics data, such as genomics, transcriptomics, metabolomics, and proteomics, where the number of variables far exceeds the number of samples [34]. By projecting patient data into principal component space, PCA facilitates the visualization of patient heterogeneity, identification of subgroups, and initial exploration of treatment response patterns. Despite its popularity and computational efficiency, PCA is inherently a linear method and may not fully capture complex non-linear relationships present in biomedical data, which has motivated the development of alternative non-linear dimensionality reduction approaches such as t-SNE, UMAP, and deep learning-based autoencoders.

### 2.3. Deep Learning

**Figure 3:**
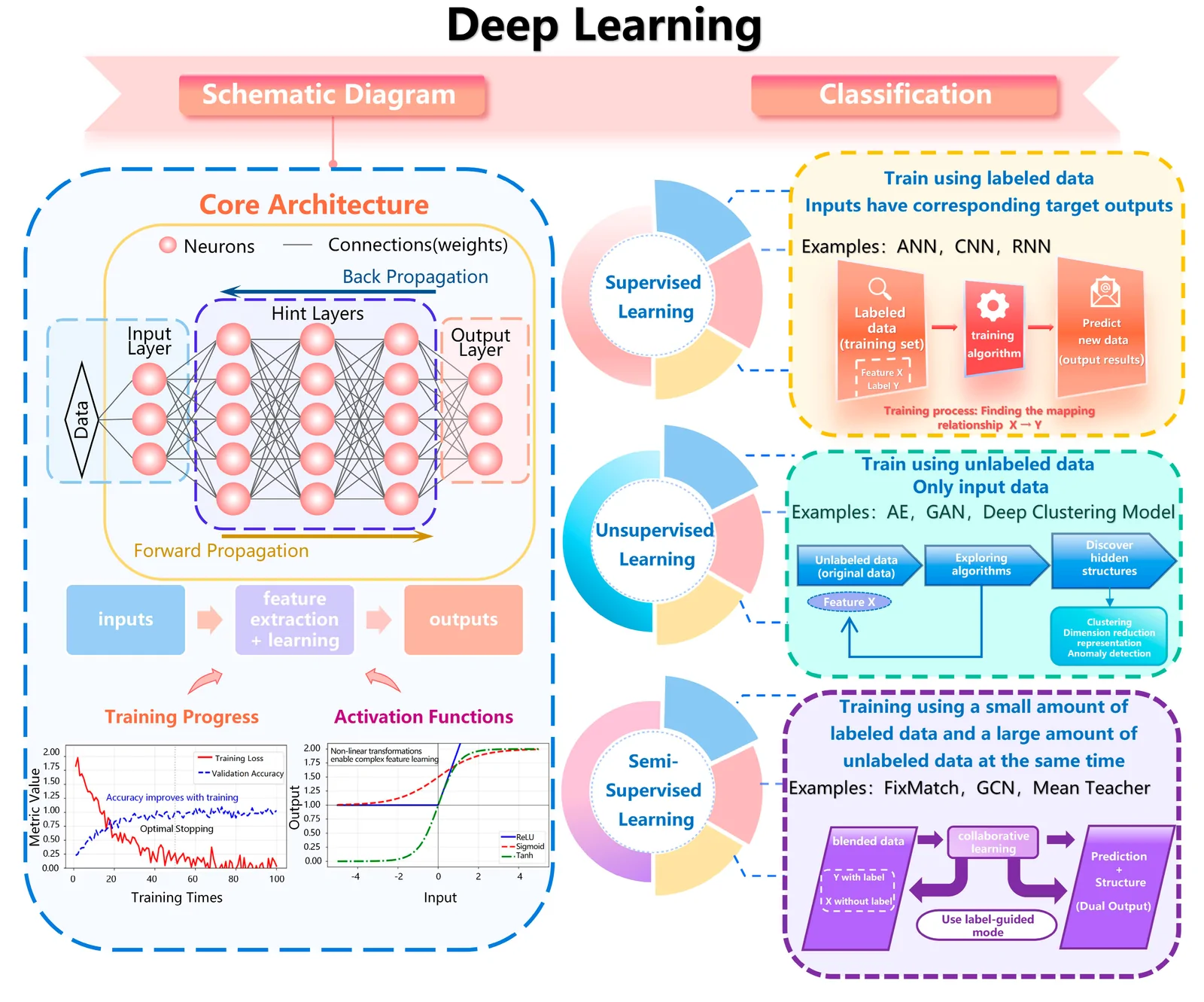
Schematic illustration of deep learning algorithms used in metabolomics analysis. Deep learning, a subset of machine learning based on multi-layer neural networks, learns hierarchical feature representations through iterative optimization. According to the availability of labeled data, DL methods are generally categorized into supervised, unsupervised, and semi-supervised learning, including artificial neural networks (ANN), convolutional neural networks (CNN), recurrent neural networks (RNN), and graph convolutional networks (GCN).

Deep Learning (DL) is a subset of ML based on neural networks (NNs) [35]. It employs multi-layer neural network architectures to progressively transform raw data into high-level abstract features. Model parameters are iteratively optimized using techniques such as regularization to enhance generalization and prevent overfitting (Figure 3) [36]. Depending on the labeling of the training data, DL algorithms can be categorized into supervised, unsupervised, and semi-supervised approaches.

Supervised DL is trained using labeled data, with commonly used architectures including artificial neural networks (ANN), convolutional neural networks (CNN), and recurrent neural networks (RNN). These models are widely applied in predictive analytics and other fields. Unsupervised DL, by contrast, focuses on learning from unlabeled data and holds significant potential for innovation in the medical field [27]. Semi-supervised learning combines a small set of labeled data with a large volume of unlabeled data, making it particularly valuable in contexts where annotated samples are limited, such as medical diagnostics. Common architectures used in semi-supervised DL include CNN, RNN, and graph convolutional networks (GCN).

In addition, neural networks with multiple hidden layers are often called deep neural networks (DNNs). They can be flexibly applied to the supervised, unsupervised, and semi-supervised DL models described above [35]. Because of its significant feature extraction capability, DL has shown great advantages in the field of precision pharmacotherapy, especially in drug efficacy prediction and drug therapeutic regimen selection.

## 3. Research Advances in Machine Learning and Metabolomics for Precision Pharmacotherapy

**Figure 4:**
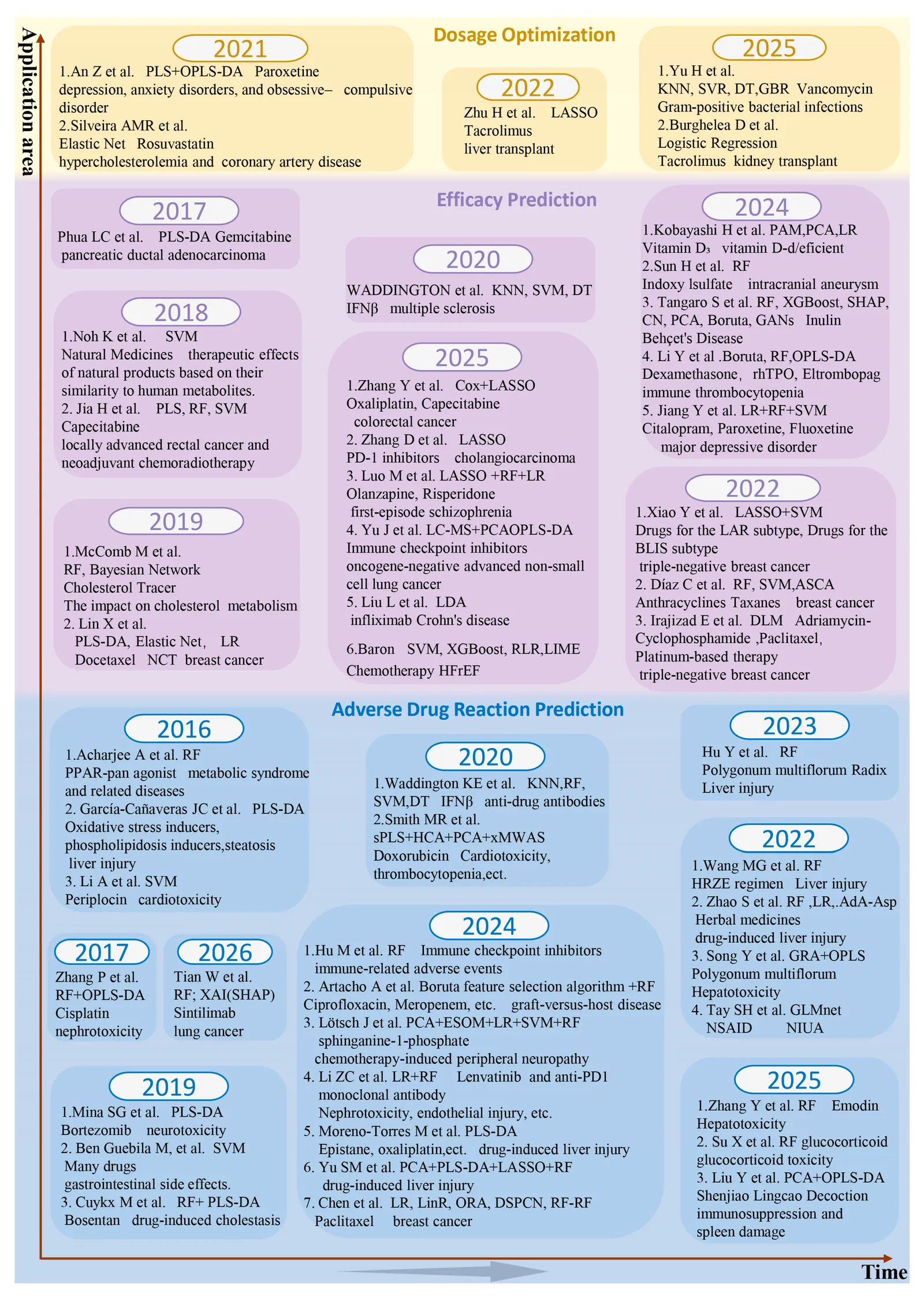
The recent applications of machine learning and metabolomics in precision pharmacotherapy. This figure summarizes studies from recent years that integrate metabolomic profiling with machine learning algorithms to support individualized medication strategies. The summarized research is systematically classified into three principal domains: (1) dose optimization, which leverages metabolic biomarkers and predictive models to tailor drug dosing. (2) Therapeutic efficacy prediction, involving metabolomics-based machine learning models that identify responders and enable personalized therapeutic decisions. (3) Adverse reaction prediction, which applies metabolomics-derived biomarkers and algorithmic risk assessment to forecast drug-induced toxicity. Together, these advances highlight the emerging role of metabolomics-driven machine learning frameworks in supporting precision medicine [37,38,39,40,41,42,43,44,45,46,47,48,49,50,51,52,53,54,55,56,57,58,59,60,61,62,63,64,65,66,67,68,69,70,71,72,73,74,75,76,77,78,79,80,81,82,83,84,85,86,87,88].

We provide an overview of the recent applications of ML-driven metabolomics in precision pharmacotherapy in Table 1 and Figure 4.

### 3.1. Applications of Metabolomics and Machine Learning in Pharmacokinetics and Dose Optimization

In current clinical practice, many medications are administered at fixed doses, overlooking inter-individual variability. For drugs with a narrow therapeutic index, where the effective dose is close to the toxic threshold, such conventional dosing strategies can lead to inconsistent efficacy and a heightened risk of adverse drug reactions. Metabolomics combined with ML algorithms has emerged as a powerful tool for building real-time, patient-specific pharmacokinetic prediction models. These models enable dynamic monitoring of drug concentrations and individualized dose adjustments, thereby maintaining therapeutic levels while minimizing toxicity. Tacrolimus, widely prescribed to maintain graft function after organ transplantation, requires precise dosing due to its narrow therapeutic window and pronounced interindividual variability. Zhu et al. [39] conducted a plasma metabolomic study in liver transplant recipients. They used the LASSO algorithm to screen out 31 endogenous metabolites associated with the dose-adjusted trough concentration (C_0_/D) of tacrolimus. A multiple linear mixed model incorporating 11 metabolites alongside clinical covariates demonstrated strong predictive performance (R_fixed_ = 0.64, R_total_ = 0.78), evaluated by 10-fold cross-validation, highlighting the value of integrating metabolomic biomarkers into dosing models. Additionally, Burghelea et al. [41] applied urinary metabolomics coupled with ML to stratify kidney transplant patients by tacrolimus exposure. A logistic regression model based on five urinary metabolites was evaluated by leave-one-out cross-validation and achieved an AUC of 0.810. These studies support the feasibility of incorporating metabolic phenotypes into tacrolimus exposure models. Traditional tacrolimus dose adjustment relies on TDM and population pharmacokinetic (PopPK) models, which are limited by univariate inputs and predefined mathematical relationships. In contrast, the integration of metabolomics and ML overcomes these limitations. However, direct comparative studies against models based on clinical covariates, TDM, PopPK, and pharmacogenomic predictors remain limited. Therefore, independent, prospective validation is required before metabolomics combined with ML algorithms can be considered suitable tools for guiding clinical dosing decisions. Furthermore, the clinical value of metabolomics combined with ML algorithms should be evaluated in terms of their incremental benefit over established TDM and PopPK approaches.

In vancomycin therapy, a critical pharmacological objective is to balance antimicrobial efficacy with the risk of nephrotoxicity by maintaining the ratio of the area under the concentration-time curve (AUC) and minimum inhibitory concentration (MIC) at less than 400 [89]. In recent years, the integration of ML techniques has introduced innovative approaches to achieving this therapeutic target. To optimize initial vancomycin dosing, Yu et al. [40] applied multiple ML algorithms using 5-fold cross-validation to predict vancomycin clearance in neonates and infants by integrating clinical and metabolomic data. The Gradient Boosting Regressor achieved high predictive accuracy (R^2^ = 0.830) using only clinical covariates. Although the addition of metabolomic features did not markedly improve predictive accuracy, several metabolites were identified as the top predictors by ML models, indicating potential mechanistic significance. However, it should be noted that the study included only 42 patients, whereas more than 4500 metabolomic features were analyzed. This pronounced imbalance between sample size and feature dimensionality increases the risk of model overfitting and may lead to optimistic estimates of predictive performance. Furthermore, no independent external validation cohort was included, and the generalizability of the model, therefore, remains uncertain. These limitations indicate that the incremental value of metabolomics for vancomycin clearance prediction requires confirmation in larger, independent cohorts with rigorous external validation.

For paroxetine, An et al. [37] demonstrated the utility of metabolomics in predicting antidepressant pharmacokinetics. By analyzing pre- and post-dose plasma samples with UPLC-HRMS, they identified 39 metabolites significantly altered by treatment. A two-stage Partial Least Squares approach linked these metabolic profiles to key PK parameters (AUC and C_max_), and a biomarker panel including cortisone, L-tyrosine, and L-valine enabled prediction of interindividual variability and discrimination between high and low responders before treatment. These findings underscore the potential of baseline metabolic signatures to guide dosing strategies and anticipate differential drug responses. However, this study included only 12 participants and lacked independent external validation. The model is at risk of overfitting, limiting confidence in the generalizability of the model.

Taken together, these studies highlight the complementary strengths of metabolomics and ML in dose optimization. Metabolomic profiling facilitates the discovery of biologically relevant features associated with drug exposure, while ML models improve predictive performance by capturing complex covariate interactions. This data-driven, individualized dosing strategy holds significant potential to transcend the limitations of traditional empirical approaches. Nevertheless, most current studies are based on small cohorts and internal validation; independent external validation and prospective clinical studies remain limited. Moreover, the incremental value of metabolomics over established approaches such as TDM, PopPK, and pharmacogenomics has not been sufficiently demonstrated. Future studies should focus on larger, prospective cohorts and direct comparative evaluation to determine whether metabolomics-informed ML models can meaningfully improve clinical dosing decisions.

### 3.2. Applications of Metabolomics and Machine Learning in Drug Efficacy Prediction

In precision pharmacotherapy, accurately predicting a drug’s efficacy for a specific patient is critical. In recent years, the integration of metabolomics with ML has emerged as a powerful strategy for individualized drug efficacy prediction, offering both mechanistic insights and translational potential. A growing body of studies across oncology, autoimmune diseases, and critical care highlights the potential of this integrative approach to uncover metabolic biomarkers, enable patient stratification, and guide therapeutic optimization. However, we need to pay particular attention to cohort size, study design, and validation strategies, as well as whether the identified markers are truly treatment-predictive rather than merely prognostic.

One important application has been in oncology, where metabolomics-guided models have revealed novel predictive signatures for chemotherapy efficacy. For instance, using pre-treatment plasma samples from 88 triple-negative breast cancer (TNBC) patients, researchers found that elevated levels of acetylated polyamines, including diacetylspermine and acetylspermidine, were associated with poorer response using deep learning, achieving high predictive accuracy (AUC = 0.97) in internal validation [50]. Similarly, lipid- and bile-acid-related metabolites were associated with response heterogeneity across breast cancer subtypes, such as triple-negative (TN), luminal B (LB), and HER2+. In TN patients, higher baseline docosahexaenoic acid (DHA) and presurgery secondary bile acids (glycodeoxycholic acid and glycohyocholic acid) were strongly associated with treatment response and overall survival, with a combined AUC of 0.946 for response prediction and 0.777 for survival prognosis, as evaluated by 100 repeated cross-validations [49]. These studies indicate the potential of integrating metabolomics with ML to predict responses to neoadjuvant chemotherapy. However, both studies were retrospective, involved relatively small sample sizes, and lacked independent external validation. These limitations may increase the risk of overfitting and reduce confidence in the generalizability of the reported predictive performance. It is also important to distinguish treatment-response prediction from survival prognosis, as these represent different clinical endpoints. Strong prognostic discrimination does not necessarily imply an ability to identify which treatment will provide the greatest benefit to an individual patient.

Beyond oncology, metabolomics has not only been applied to predict interindividual variation in drug pharmacokinetics but also directly to predict interindividual differences in drug efficacy. In immune-mediated diseases, metabolomic-ML approaches provided insights into treatment resistance in immune thrombocytopenia (ITP), identifying lipid metabolism and mitochondrial dysfunction as key contributors [54]. This finding suggests that drugs targeting lipid metabolism or mitochondrial function could be prospectively explored as a combination therapy strategy for patients with treatment-resistant ITP. Simultaneously, relevant metabolites could serve as biomarkers for identifying suitable candidates among patients with resistant disease. Similarly, in Behçet’s disease, integration of microbiome, metabolome, and XAI was proposed as a framework for individualized interventions [53]. This framework can identify distinct patient metabolic subgroups through cluster analysis and use XAI to provide interpretable evidence for drug efficacy prediction. Notably, an observational study in intracranial aneurysms [52] and a randomized, placebo-controlled VITdAL-ICU trial [51] in intracranial aneurysms and critical illness demonstrated the broader clinical value of metabolic stratification: gut microbiome-derived metabolites such as indoxyl sulfate were implicated in aneurysm rupture risk, while metabolic clustering predicted differential survival benefits from vitamin D supplementation in ICU patients. Although the former study used an independent external validation cohort and achieved an AUC of 0.972, it still requires further validation through prospective studies. The latter study was a post hoc analysis of a randomized controlled trial, which validated cluster stability only through sensitivity analysis and did not include an independent external cohort, so its conclusions require further validation using an independent external cohort.

Taken together, current studies demonstrate the potential of metabolomics combined with ML algorithms for treatment response prediction, but the available evidence remains at an exploratory stage. Many models are derived from relatively small or retrospective cohorts, and independent external or prospective validation remains limited. More importantly, most studies focus on distinguishing responders from non-responders within a single treatment regimen, rather than determining which of several available therapies would provide the greatest benefit for an individual patient. Future research should prioritize externally validated and prospective models that compare alternative treatment options, assess the incremental value of metabolomic features beyond established clinical predictors, and distinguish treatment-predictive biomarkers from general prognostic markers. Such studies will be essential before metabolomics-based ML models can provide robust support for individualized treatment selection.

### 3.3. Applications of Metabolomics and Machine Learning in Adverse Drug Reaction Prediction

Adverse drug reactions (ADRs) are harmful and unintended responses to drugs administered at normal doses, unrelated to their therapeutic purpose [90]. They may arise from a single drug or from drug–drug interactions, often necessitating dose reduction or discontinuation and ultimately leading to treatment failure. Accurate prediction of ADRs is therefore critical to ensuring rational drug use. ML-integrated metabolomics offers a potential strategy for identifying multivariate metabolic features associated with ADRs. However, the extent to which these models improve prediction beyond conventional clinical, exposure-based, or pharmacogenomic risk factors remains incompletely established.

Natural products such as periplocin, *Polygonum multiflorum*, emodin, and paclitaxel are widely used in clinical practice but are frequently associated with severe ADRs, particularly cardiotoxicity and drug-induced liver injury. Recent studies have applied metabolomics in combination with ML to systematically evaluate their safety and elucidate toxicity mechanisms. For example, Li et al. [67] utilized ultra-performance liquid chromatography coupled with quadrupole time-of-flight mass spectrometry (UPLC-Q-TOF/MS) and SVM to identify 11 cardiotoxicity-associated biomarkers of periplocin. The SVM model achieved 87.5% accuracy during cross-validation and 100% accuracy in an internally partitioned hold-out test set. However, the absence of an independent external cohort and the use of primary cardiomyocytes from a single experimental batch limit confidence in the robustness and generalizability of the reported performance.

In liver injury caused by Polygonum multiflorum, Hu et al. [77] analyzed clinical plasma metabolomic profiles with an RF model and identified three highly sensitive and specific biomarkers for early ADR prediction, which can be used for early warning of liver toxicity during treatment with Polygonum multiflorum. Notably, this study only enrolled 6 positive liver injury cases and lacked independent external validation and prospective cohort verification, so the clinical utility of the biomarkers remains to be confirmed by larger cohorts. Similarly, Song et al. [75] employed a back-propagation ANN with internal validation to evaluate model performance and identified three hepatotoxic constituents of Polygonum multiflorum, including emodin. This workflow can be used for toxicity screening of lead compounds during the drug development process to eliminate high-risk candidate molecules, while Zhang et al. [85] integrated LC-MS and NMR with a double random forest (RF-RF) classifier to identify 17 valid differential metabolites linked to hepatotoxicity, which may provide candidate biomarkers for future clinical risk stratification and safety monitoring. In contrast to studies reporting high classification performance, Chen et al. [84] used ML to identify metabolic biomarkers of paclitaxel neurotoxicity among 20 amino acids, but the model performed poorly. This highlights that the key to the successful application of ML lies in high-quality feature inputs, meaning that the predictive value of ML depends strongly on whether the measured features contain sufficient information related to the outcome. In Chen’s research, amino acids such as glutamate and phenylalanine showed only weak statistical associations with neurotoxicity, suggesting that the available metabolic feature space may not have captured sufficient biological information for reliable prediction. These studies illustrate the potential of integrating ML with metabolomics for toxicity-related biomarker discovery and early risk assessment. Rigorous external validation, representative sampling, and solid biological grounding between input features and tested outcomes are core requirements for the successful application of this approach.

For small-molecule drugs such as cisplatin and doxorubicin, severe ADRs, including nephrotoxicity, cardiotoxicity, and thrombocytopenia, remain major clinical concerns. Zhang et al. [68] applied non-targeted metabolomics and ML to rat kidney tissues and revealed pronounced medullary toxicity of cisplatin, identifying 29 key metabolites associated with nephrotoxicity. Macromolecular drugs such as interferon-β (IFNβ) and immune checkpoint inhibitors (ICIs) also exhibit significant safety risks due to anti-drug antibody (ADA) formation and immune-related adverse events (irAEs). Approximately 35% of multiple sclerosis (MS) patients develop ADA following IFNβ therapy. Waddington et al. [47] employed serum metabolomics combined with six ML classifiers to predict ADA development, which were all evaluated via ten-fold cross-validation, where lasso logistic regression achieved high predictive accuracy (F1 = 0.808, specificity = 0.91). Yet a limited sample size hindered stratified analysis, and dosage forms introduced confounding bias. In the context of ICIs, Hu et al. [78] developed an RF classifier based on 14 microbial features to predict irAEs. The classifier was evaluated via stratified ten-fold cross-validation, study-to-study transfer, and leave-one-study-out validation, as well as on two retrospective independent cohorts, achieving an AUC of 0.88 across multiple cohorts. Targeted metabolomics further revealed that irAE-free patients exhibited significantly higher serum methylnaphthoquinone levels than affected patients, underscoring the utility of microbial and metabolic biomarkers in predicting irAE onset and guiding early intervention. Nevertheless, its performance was constrained by confounders, including tumor subtypes and treatment regimens, and real-world prospective validation remains lacking. While most existing studies focus on ADR prediction for single-drug regimens, research is increasingly extending to combination therapies. Li et al. [81] constructed an ML model based on metabolomic signatures to identify plasma features associated with ADRs in patients receiving Lenvatinib combined with anti-PD-1 therapy for unresectable hepatocellular carcinoma, built with repeated ten-fold cross-validation for internal assessment and further validated via independent external validation, revealing underlying metabolic mechanisms of drug synergy and toxicity. Wang et al. [73] further demonstrated that pre-treatment metabolomic and microbiome profiling, combined with multiple ML models, could achieve accurate early prediction of anti-tuberculosis drug-induced liver injury, which was tuned and assessed via repeated five-fold cross-validation, offering a novel ADR warning system for clinical use. However, the model performance was evaluated mainly within the studied cohorts, and its generalizability requires further independent external validation and prospective verification.

Overall, current evidence suggests that metabolomics integrated with ML has large potential for ADR risk stratification, biomarker discovery, and mechanistic investigation. However, the level of evidence remains heterogeneous. Many studies rely on small or retrospective cohorts, internal validation, or experimental toxicity models, and only a limited number have undergone independent external validation. The key translational gap is therefore not simply achieving high discrimination but demonstrating reproducible performance and incremental clinical value across independent populations and real-world treatment settings. Future studies should prioritize multicenter prospective validation and assess whether metabolomics-informed models improve ADR prevention or treatment decisions beyond established clinical, pharmacokinetic, and pharmacogenomic risk factors.

## 4. Key Barriers to Clinical Translation of ML-Integrated Metabolomics

**Figure 5:**
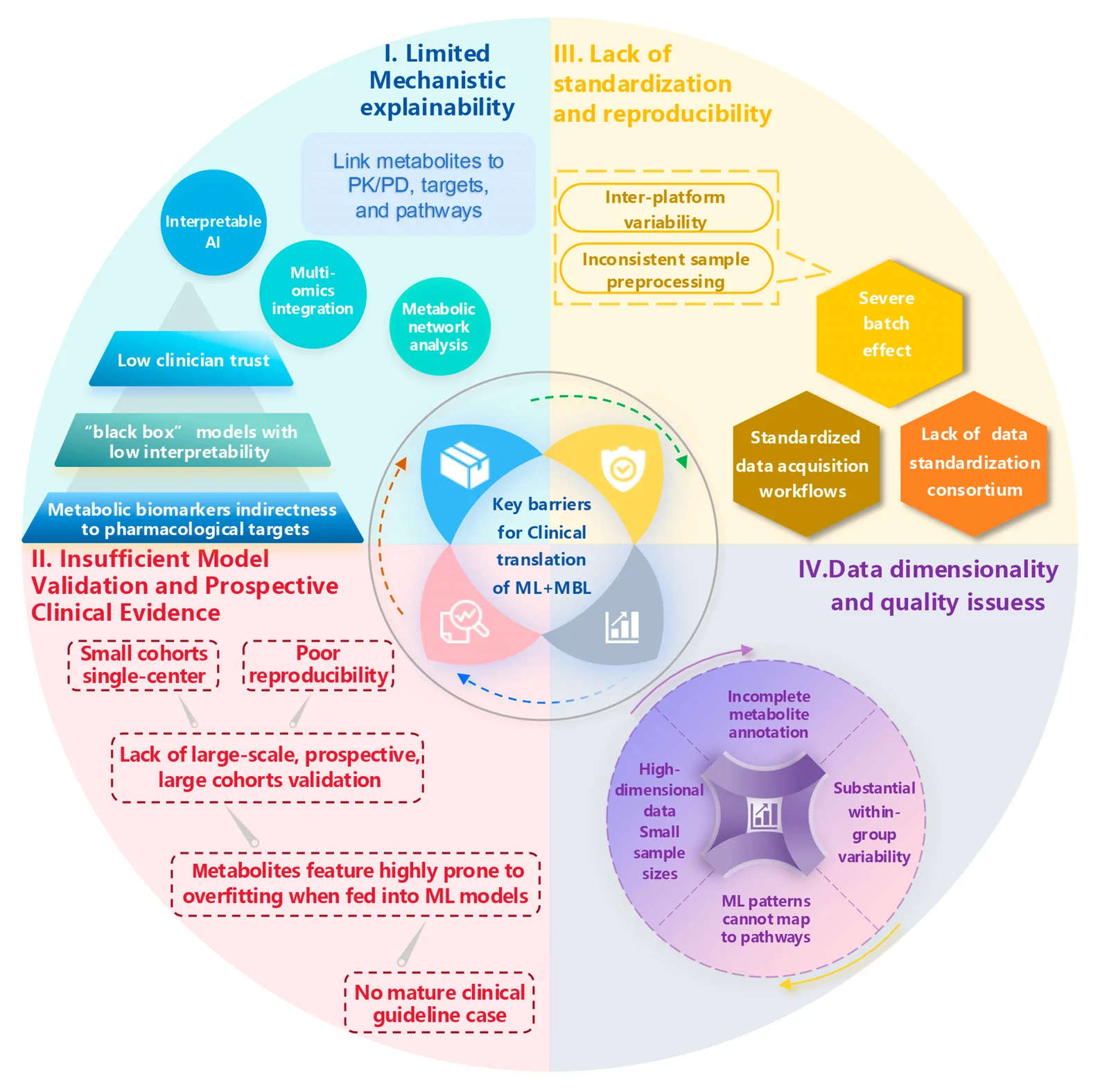
Key barriers and potential strategies for the clinical translation of ML-integrated metabolomics. The major challenges include limited explainability, insufficient prospective clinical validation, lack of standardization and reproducibility, and data dimensionality and quality issues.

ML integrated with metabolomics has made rapid progress in precision pharmacotherapy over recent years. While these advances hold significant promise, the translation of ML integrated with metabolomics into routine clinical practice remains hindered by substantial challenges. The high dimensionality, strong correlations, and incomplete metabolite annotation in metabolomics aggravate critical ML challenges such as interpretability and overfitting. Meanwhile, ML algorithms’ decision-mechanism dependence and feature-importance biases severely undermine biomarker discovery’s stability and reproducibility. The limited clinical translation of metabolomics integrated with ML is primarily constrained by four key challenges: limited mechanistic explainability, insufficient prospective clinical validation, lack of standardization and reproducibility, and data dimensionality and quality issues. This section provides a critical assessment of four major challenges and discusses future directions for the application of ML integrated with metabolomics in precision pharmacotherapy (Figure 5).

### 4.1. Limited Mechanistic Explainability

Many ML models, particularly DL, are regarded as “black-box” models with low interpretability because their internal decision-making logic is complex and the underlying operations are difficult to visualize or explicitly demonstrate. The biological rationale behind their predictions is not easily elucidated, which diminishes clinicians’ trust [91]. On the other hand, metabolomics measures downstream small-molecule metabolites, whereas the efficacy or toxicity targets of most drugs reside upstream at the protein level (such as enzymes, receptors, and ion channels). Multiple layers of metabolic regulatory networks lie between the two, making it difficult for changes in metabolite levels to directly and specifically reflect the functional state of the target [92]. This indirectness further complicates the biological interpretation of associations between metabolite features and drug mechanisms of action. In summary, the low interpretability of ML and the indirect nature of metabolic markers jointly result in the poor transparency of predictions derived from integrating ML and metabolomics, severely limiting the practical value of this technology in clinical applications.

To address this dual challenge, three complementary strategies proposed in recent years deserve attention. Firstly, multi-omics integration can help bridge the gap between downstream metabolite changes and their upstream protein executors. Secondly, metabolic network analysis can infer the partial order relations of metabolite interactions from steady-state observational data [93]. Thirdly, XAI methods partially alleviate the “black box” problem by quantifying the marginal contribution of each metabolic feature to the model output [91]. In the reviewed studies, specific XAI techniques, including SHAP, LIME, and attention mechanisms, have been increasingly adopted. For instance, SHAP analysis has been applied to identify key metabolites contributing to treatment response predictions in breast cancer [50], while LIME has been used to provide patient-level explanations for metabolic syndrome risk prediction [91]. These methods provide both global feature importance rankings and local explanations for individual predictions, thereby improving model transparency and facilitating the discovery of biologically interpretable metabolite signatures. Importantly, however, XAI explains how a model generates its predictions but does not by itself establish biological causality. Therefore, XAI should ideally be combined with pathway analysis, multi-omics evidence, and experimental validation to improve both computational and biological interpretability.

### 4.2. Insufficient Model Validation and Prospective Clinical Evidence

Currently, most studies integrating ML with metabolomics remain retrospective and exploratory, with generally small cohorts and relying predominantly on internal validation. Internal resampling approaches, including k-fold cross-validation, repeated cross-validation, and bootstrap resampling, are important for estimating model performance during development but cannot establish generalizability to independent populations. Moreover, when feature selection and hyperparameter optimization are performed, these procedures should be incorporated within the resampling framework to minimize information leakage and optimistic performance estimates. Independent external validation, from different centers, populations, and analytical batches, therefore provides a substantially stronger test of model transportability. Beyond discrimination, model calibration should also be evaluated to determine whether predicted probabilities correspond appropriately to observed outcome frequencies. Calibration plots and calibration intercepts and slopes should be reported, while Brier scores can provide an additional measure of overall probabilistic accuracy. Transparent reporting is equally important: studies should clearly document cohort selection, data preprocessing, missing-data handling, feature-selection procedures, hyperparameter tuning, validation design, and all reported performance measures in accordance with established reporting guidance such as TRIPOD + AI [94]. PROBAST + AI may further support the assessment of methodological quality, risk of bias, and clinical applicability [95].

In addition, a lack of large-scale, prospective cohort validation undermines the credibility of the findings and their clinical translatability. Most published studies focus on discrimination performance rather than whether model-guided decisions improve patient outcomes. An important question for precision pharmacotherapy is whether metabolomics-based ML models provide meaningful incremental value beyond established approaches such as clinical risk factors, TDM, PopPK, PK/PD modeling, and pharmacogenomics. Improvements in AUROC or accuracy alone may not justify clinical implementation unless they translate into more appropriate dosing, improved efficacy, reduced toxicity, or other measurable patient benefits. NICE has issued multiple relevant clinical guidelines, and the FDA has approved over 950 AI/ML-enabled medical devices in the field of medical imaging [96]. No mature clinical guidelines or implementation frameworks have yet emerged for ML-integrated metabolomics. Therefore, the research paradigm must shift decisively from current small cohorts, and retrospective studies toward prospective, multicenter, large-cohort investigations that rigorously validate biomarkers with clinical translation potential. Finally, even when adequate predictive and clinical validity has been demonstrated, regulatory, clinical workflow integration, and cost-effectiveness remain important implementation considerations [97].

### 4.3. Lack of Standardization and Reproducibility

The lack of standardization in sample collection, analytical measurement, data processing, and reporting workflows substantially limits the cross-laboratory comparison and validation of results, making it difficult to reach consensus even across studies of identical biological samples. At the core of this challenge is the lack of harmonization across different analytical platforms, as the instrument models, column types, and data acquisition modes employed by distinct platforms such as liquid chromatography-mass spectrometry (LC-MS) and gas chromatography-mass spectrometry (GC-MS) vary considerably, resulting in data with highly heterogeneous technical characteristics. In addition, pre-analytical factors, including fasting status, sample collection procedures, storage conditions, and freeze–thaw cycles, can substantially influence metabolite profiles and introduce additional sources of variability. Furthermore, batch effects caused by instrument signal drift, reagent lot differences, and variations in laboratory operating conditions may introduce systematic errors that can easily be misinterpreted as genuine biological signals. Notably, models developed under batch-confounded datasets may show apparently strong internal performance but deteriorate when applied to independently generated datasets.

Overcoming this bottleneck requires establishing a metabolomics data standardization consortium to coordinate the construction of standardized metabolomics protocols. Existing mature public platforms can play a key supporting role. For instance, MetaboAnalyst provides standardized workflows for metabolomics data processing and multi-omics integrative analysis [6]. The Metabolomics Innovation Centre (TMIC) offers comprehensive metabolomics platforms integrating advanced analytical technologies, standardized workflows, and extensive database resources. Collectively, these resources facilitate greater harmonization of metabolomics workflows, data processing, and metabolite annotation, although broader interlaboratory standards and validation efforts remain necessary.

### 4.4. Data Dimensionality and Quality Issues

Finally, high-dimensional data, small sample sizes, and low metabolite annotation coverage severely limit the clinical applicability of ML–metabolomics integration. At the data level, metabolomics faces several fundamental challenges. Firstly, metabolomics datasets often contain thousands of metabolic features, whereas clinical sample sizes remain relatively limited. This “high-dimensional, small-sample” structure, combined with substantial interindividual biological heterogeneity and strong correlations among metabolites, substantially increases the risk of overfitting and unstable feature selection. Secondly, in untargeted metabolomics, only 2–20% [98] of metabolic features can be successfully annotated with known chemical structures, and the vast majority of detected signals correspond to “unknown metabolites.” Although substantial progress has been made through the development of comprehensive databases and analytical platforms, such as HMDB and METLIN, challenges persist in accurately identifying and quantifying metabolites across diverse experimental settings. As a result, the large proportion of unidentified or ambiguously annotated features limits pathway-level interpretation and complicates the translation of ML-selected signals into biologically interpretable and clinically measurable biomarkers.

Beyond these structural issues, missing data, normalization handling, and feature selection constitute additional challenges at the data level that critically impact machine learning performance. Due to detection limits and spectral peak overlap, missing values are prevalent in metabolomics data. Importantly, missing values in metabolomics are not random, and inappropriate imputation may distort metabolite distributions and downstream ML performance [99]. Similarly, no universally optimal normalization strategy exists. Inappropriate normalization methods can distort the relative abundance of metabolites, introduce spurious differences, alter relationships among features, and ultimately compromise the robustness and reproducibility of model-based results. Furthermore, feature selection represents another major source of model instability because highly correlated metabolites can produce different but apparently equivalent feature subsets across resampled datasets. If researchers treat feature selection merely as a standardized preprocessing step without conducting robustness validation, they may derive poorly reproducible prediction models and identify spurious metabolic biomarkers [100]. Feature selection and hyperparameter tuning should instead be conducted within each training fold, or within an appropriate nested resampling framework, to minimize information leakage.

Addressing these challenges requires not only improved experimental design and data acquisition but also the development of advanced computational strategies tailored to high-dimensional, noisy datasets. Such practices are essential for distinguishing reproducible metabolic biomarkers from dataset-specific predictive signals.

## 5. Conclusions

The integration of ML and metabolomics offers a powerful framework by linking genetic variation to downstream biochemical phenotypes and enabling data-driven prediction of drug exposure, therapeutic efficacy, and toxicity. However, challenges including limited explainability, insufficient model validation and prospective clinical evidence, lack of standardization and reproducibility, and high-dimensional data continue to hinder clinical translation. In the near term, one of the most clinically feasible applications of ML–metabolomics is likely dose optimization for narrow-therapeutic-index drugs. Future research should focus on high-quality, standardized datasets and interpretable and robust models. In particular, it is necessary to strengthen the integration of multi-omics, such as metabolomics, genomics, and proteomics, to reveal the complex mechanisms underlying drug responses and provide more reliable decision support for precision pharmacotherapy. Ultimately, the clinical value of ML-integrated metabolomics will depend not only on predictive accuracy but also on whether it provides reproducible and clinically meaningful incremental benefit beyond established pharmacological and clinical decision-making approaches.

## Figures and Tables

**Table 1:** The overview of the recent applications of machine learning and metabolomics in precision pharmacotherapy.

Application Area	Author(s)	Year of Publication	Medicine	Model Name/Type	Key Point
Pharmacokinetics and Dose Optimization	An Z et al. [37]	2021	Paroxetine	Two-stage Partial Least Squares; predicts individual drug response (high vs. low responders). AUC: R^2^Y = 0.799, Q^2^ = 0.678; C_max_: R^2^Y = 0.710, Q^2^ = 0.551	Paroxetine is used in the therapy of depression, anxiety disorders, and obsessive–compulsive disorder.
	Silveira AMR et al. [38]	2021	Rosuvastatin	Elastic Net (EN) (ESI + mode): Training set R^2^ = 1.00, cross-validation R^2^ = 0.93, RMSE = 19.03 ng·h/mL, MAPE = 10.12%	Rosuvastatin is used to treat hypercholesterolemia and prevent coronary artery disease.
	Zhu H et al. [39]	2022	Tacrolimus	LASSO: MAE = 0.611, RMSE = 0.788, R = 0.78	Tacrolimus is used for immunosuppressive therapy in liver transplant recipients.
	Yu H et al. [40]	2025	Vancomycin	KNN, SVR, Decision Tree (DT), Gradient Boosting Regressor (GBR), RFR, AdaBoost, XGBoost, Logistic Regression, LASSO. GBR combined with 10 clinical covariates: MSE = 0.0033, R^2^ = 0.830	Vancomycin is used to treat suspected or confirmed Gram-positive bacterial infections.
	Burghelea D et al. [41]	2025	Tacrolimus	Logistic Regression: AUC = 0.810, CA = 0.690	Tacrolimus is used for immunosuppressive therapy in kidney transplant patients.
Drug Efficacy	Phua LC et al. [42]	2017	Gemcitabine	PLS-DA: AUC = 1.0	Gemcitabine is used for the adjuvant chemotherapy of pancreatic ductal adenocarcinoma, and the partial least squares discriminant analysis (PLS-DA) model can accurately distinguish patients with different treatment responses.
	Jia H et al. [43]	2018	Capecitabine (combined with radiotherapy)	PLS: AUC = 0.87, RF: AUC = 0.83, SVM: AUC = 0.85	Metabolic biomarkers screened through machine learning models can effectively distinguish between patients with locally advanced rectal cancer who are sensitive to capecitabine-based neoadjuvant chemoradiotherapy and those who are resistant to it.
	Noh K et al. [44]	2018	Natural Medicines	SVM: AUROC = 0.893	The study proposes a systematic approach to predicting the therapeutic effects of natural products based on their similarity to human metabolites.
	McComb M et al. [45]	2019	Cholesterol Tracer	RF: PR = 0.853, M_1_ = 0.821, M_3_ = 0.780, M_tot_ = 0.845; Bayesian Network	By outputting the core parameters of cholesterol metabolism through the model, it can simulate the impact of drugs on cholesterol metabolism, thereby providing a quantitative basis for dosage optimization and efficacy prediction of drugs in diverse populations.
	Lin X et al. [46]	2019	Epirubicin, Cyclophosphamide, Docetaxel, Trastuzumab	PLS-DA, EN, Logistic Regression: AUC = 0.957, Specificity = 100%, Sensitivity = 81.2%	A therapeutic effect prediction model for breast cancer treated with an anthracycline-docetaxel-based neoadjuvant chemotherapy regimen was constructed based on 9 serum metabolites.
	Waddington KE et al. [47]	2020	IFNβ	KNN, SVM, DT: F1 = 0.778, Specificity = 0.942, Classification accuracy = 0.854; LASSO, Logistic Regression, Lasso with Interactions	Patients with multiple sclerosis may develop IFNβ ADA, which impairs drug efficacy. Machine learning models have been established based on serum metabolomics data to predict the development of ADA, aiming to identify patients at risk of treatment failure prior to therapy.
	Xiao Y et al. [48]	2022	Drugs for the LAR subtype, Drugs for the BLIS subtype	LASSO: AUC = 0.9549; SVM	The study focuses on the precision medicine of TNBC, discovers potential therapeutic targets, and establishes machine learning models for subtype stratification.
	Díaz C et al. [49]	2022	Anthracyclines, Taxanes	RF, SVM, ANOVA-Simultaneous Component Analysis (ASCA): Efficacy prediction (response vs. non-response): AUC = 0.946; Prognosis prediction: AUC = 0.777	A model is established that can effectively analyze the association between metabolomics data and the treatment response of breast cancer treated with anthracycline-taxane-based neoadjuvant chemotherapy (NACT).
	Irajizad E et al. [50]	2022	Adriamycin-Cyclophosphamide, Paclitaxel, and Platinum-based therapy	DL: Baseline prediction (distinguishing RCB-II/III vs. RCB-0/I), AUC = 0.97	Effectively predict the treatment response of TNBC treated with NACT.
	Kobayashi H et al. [51]	2024	Vitamin D_3_	Partitioning Around Medoids: Consistency of the original clustering results > 75%; LR, PCA: R^2^X (cum) = 0.718	Only vitamin D-deficient patients with specific metabolic phenotypes derive significant benefits from high-dose vitamin D_3_ treatment, and a model is established to effectively distinguish metabolic phenotypes and predict treatment responses.
	Sun H et al. [52]	2024	Antibiotic cocktail, Indoxyl sulfate	RF: Test set AUC = 0.974	Reveals the association between gut microbiota-derived metabolites (such as indoxyl sulfate, IS) and the progression/rupture of IA.
	Tangaro S et al. [53]	2024	Inulin	RF, XGBoost, SHAP, CN, PCA, Boruta, Generative Adversarial Networks (GANs)	Investigate the relationships between the microbiome, volatilome (volatile subset of metabolome), and clinical characteristics in patients with Behçet’s Disease, and elucidate the pathogenesis and improve clinical outcomes through data-driven, explainable artificial intelligence models.
	Li Y et al. [54]	2024	Dexamethasone, rhTPO, Eltrombopag	Boruta, RF, OPLS-DA: VIP > 1, *p* < 0.05	Five treatment regimens, including dexamethasone, recombinant human thrombopoietin (rhTPO), and eltrombopag, are used to treat ITP. Metabolic biomarkers are screened through models, which can effectively distinguish between patients with treatment response and non-response.
	Jiang Y et al. [55]	2024	Citalopram, Paroxetine, Fluoxetine	Logistic Regression: Training set AUC = 0.993; RF, SVM	Selective serotonin reuptake inhibitors are used to treat major depressive disorder, and three machine learning models are employed to predict treatment response based on gut microbiota and metabolite characteristics.
	Zheng L et al. [56]	2025	PD-1 inhibitor combined with chemotherapeutic agents	LASSO, RF: Training set AUC = 0.973, validation set AUC = 0.944; SVM, Logistic Regression	Chemoimmunotherapy combining PD-1 inhibitors with chemotherapy is used to treat advanced lung squamous cell carcinoma, and a model is constructed to effectively distinguish between responders and non-responders, as well as high-risk and low-risk populations.
	Zhang J et al. [57]	2025	Cisplatin	CoxBoost, Stepwise Cox Regression, EN, Gradient Boosting, LASSO, Partial Least Squares, Cox, Random Survival Forest, Ridge, Supervised Principal Components, survival-SVMs	A B cell-associated Scissor+ related B cell score model has been developed for lung adenocarcinoma, which can effectively stratify patient risk and predict treatment response.
	Buck A et al. [58]	2022	neoadjuvant therapy	RF: HR = 3.38, *p* = 0.007	The study focuses on esophageal adenocarcinoma, and the neoadjuvant treatment regimens used include platinum/5-fluorouracil (5-FU) chemotherapy and 45Gy radiotherapy combined with platinum/5-FU chemoradiotherapy.
	Zhang Y et al. [59]	2025	Oxaliplatin, Capecitabine	Cox: Training set, 1-year RFS prediction AUC = 0.959; LASSO	The XELOX regimen (oxaliplatin combined with capecitabine) is used to treat colorectal cancer, and a predictive model is constructed to effectively predict the chemosensitivity of XELOX.
	Zhang D et al. [60]	2025	PD-1 inhibitors	LASSO: Training set R^2^ = 0.83	Integrating biliary metabolomic data and the Immune Hot-Cold Index through the LASSO regression model enables effective prediction of treatment response to PD-1 inhibitors in cholangiocarcinoma patients.
	Luo M et al. [61]	2025	Olanzapine, Risperidone, etc.	LASSO, RF, Logistic Regression: AUC = 0.805	Five antipsychotic drugs, including olanzapine and risperidone, are used to treat first-episode schizophrenia. By constructing a model, 3 core lipid biomarkers are screened out, which can effectively distinguish between patients with treatment response and non-response.
	Baron C et al. [62]	2025	Heart failure classification	SVM, XGBoost, Ridge Logistic Regression, LIME	This study analyzed 55 plasma metabolites and used SVM and XGBoost to identify lignoceric acid as a critical discriminator for distinguishing patients with HFrEF from the control group. The SVM achieved an accuracy of 85.73%, while XGBoost achieved 84.8%, and these results were validated in a replication cohort. LIME was used to assess local interpretability for individual predictions.
	Yu J et al. [63]	2025	Immune checkpoint inhibitors	MLR, OPLS-DA: ESI + mode, R^2^Y = 0.811, Q^2^ = 0.465	ICIs combined with chemotherapy are used as the first-line regimen for treating oncogene-negative advanced non-small cell lung cancer. Through a multivariate logistic regression model, 3 core lipid biomarkers are screened out, which can effectively distinguish between patients with treatment response and non-response.
	Liu L et al. [64]	2025	infliximab	LDA: Test set AUC = 0.805; RF, etc.	Infliximab is used to treat Crohn’s disease, and the gut microbiota-based Linear Discriminant Analysis (LDA) model can effectively distinguish between patients with treatment response and non-response.
Adverse Drug Reaction Prediction	Acharjee A et al. [65]	2016	PPAR-pan agonist	RF: Variance explanation rate, Q^2^ = 84%	PPAR pan-agonists are used in the treatment of metabolic syndrome and related diseases (such as insulin resistance, dyslipidemia, etc.).
	García-Cañaveras JC et al. [66]	2016	Oxidative stress inducers, phospholipidosis inducers, and steatosis inducers	PLS-DA: R^2^ = 0.832, Q^2^ = 0.686	By using a variety of known hepatotoxic compounds (to simulate different mechanisms of liver injury), a partial least squares discriminant analysis [PLS-DA(R^2^ = 0.832, Q^2^ = 0.686)] model was established to predict drug-induced liver injury and classify its injury mechanisms.
	Li A et al. [67]	2016	Periplocin	SVM: cross-validation accuracy = 87.5%; independent test set predictive accuracy = 100%	Periplocin is used for the treatment of rheumatoid arthritis and chronic congestive heart failure, but it is prone to causing cardiotoxicity.
	Zhang P et al. [68]	2017	Cisplatin	RF, OPLS-DA: In the renal medulla, the Q^2^ values of the low, medium, and high dose groups are 0.45, 0.872, and 0.949, respectively	Cisplatin is used to treat a variety of solid tumors but is prone to causing nephrotoxicity. A model has been established in the study to reveal the difference in sensitivity to cisplatin between the renal cortex and medulla.
	Mina SG et al. [69]	2019	Bortezomib	PLS-DA: Bortezomib group R^2^ = 0.97, Q^2^ = 0.79; LASSO: Goodness of fit for predicting cumulative DJ-1 levels R^2^ = 0.88	Bortezomib is used to treat relapsed/refractory multiple myeloma and mantle cell lymphoma but has neurotoxicity.
	Ben Guebila M et al. [70]	2019	Many drugs	SVM Combined feature model: Micro-average AUROC = 0.94	The study did not focus on the correspondence between specific therapeutic drugs and diseases. Instead, it covered a variety of marketed small-molecule drugs and established a multi-label SVM to predict drug-induced gastrointestinal side effects.
	Cuykx M et al. [71]	2019	Bosentan	PLS-DA, RF: 24 h exposure group Q^2^ > 0.6	Bosentan is used for the treatment of pulmonary arterial hypertension but is prone to causing drug-induced cholestasis. A study established models to screen for metabolic biomarkers associated with cholestasis.
	Waddington KE et al. [47]	2020	Beta interferons	KNN, RF, SVM, DT: F1 score = 0.778, specificity = 0.942, classification accuracy = 0.854; LLR + I, LASSO logistic regression: F1 = 0.808, specificity = 0.91	IFNβ is used for the treatment of RRMS and CIS, but some patients may develop ADA. The study established predictive models based on baseline serum metabolomics to identify patients at high risk for ADA development prior to IFNβ treatment, helping avoid subsequent efficacy decline caused by ADA.
	Smith MR et al. [72]	2020	Doxorubicin	xMWAS, PCA, Hierarchical Cluster Analysis (HCA): FDR < 0.2	Doxorubicin (Adriamycin, Dox) is used for the treatment of various cancers such as leukemia, multiple myeloma, and breast cancer, but it is prone to causing side effects like cardiotoxicity and thrombocytopenia. A study established models to integrate platelet metabolomics and bioenergetics data, revealing the drug’s impact on the metabolic-energetic interaction network.
	Wang MG et al. [73]	2022	HRZE regimen (INH + RFP + PZA + EMB)	RF: AUC = 0.98; ANN, SVM-linear, SVM-rbf	First-line anti-tuberculosis drugs are used for the treatment of tuberculosis, and models have been established to predict the risk of drug-induced liver injury.
	Zhao S et al. [74]	2022	Herbal medicines	RF, Logistic Regression, AdA-Asp: Training set performance AUC = 0.889, sensitivity = 73.7%, specificity = 92.7%	To evaluate the risk of chronicity of drug-induced liver injury (DILI) that may be caused by a variety of drugs (including herbal medicines, conventional drugs, etc.) when they are used to treat relevant diseases.
	Song Y et al. [75]	2022	Polygonum multiflorum	GRA, OPLS: Model parameters of L02 cells R^2^X = 0.94, R^2^Y = 0.82, Q^2^ = 0.67; BP-ANN: L02 cell model training set R = 0.938	The study aims to screen for potential hepatotoxic components in raw Polygonum multiflorum, which exerts the effects of detoxification, resolving carbuncles, and moistening the intestines to relieve constipation.
	Tay SH et al. [76]	2022	Nonsteroidal anti-inflammatory drugs	GLMnet: L02 cell model training set R = 0.938	The model distinguishes between the pre-desensitization and post-desensitization states of patients with nonsteroidal anti-inflammatory drug-induced urticaria/angioedema and the state of healthy controls.
	Hu Y et al. [77]	2023	Polygonum multiflorum Radix	RF-ROC: Three key biomarkers: hypoxanthine (AUC = 0.974, Sen = 1.000, Spe = 0.846), LysoPC (P-16:0/0:0) (AUC = 1.000, Sen = 1.000, Spe = 1.000), and taurochenodesoxycholic acid (AUC = 0.974, Sen = 1.000, Spe = 0.846)	The study screened for biomarkers of liver injury induced by Polygonum multiflorum (which exerts the effects of tonifying the liver and kidneys and lowering blood lipids) using algorithms and established a diagnostic model based on 3 core biomarkers.
	Hu M et al. [78]	2024	Immune checkpoint inhibitors	RF: distinguish between irAEs and non-irAEs. Average AUC = 0.88	The study established a classification model to predict the risk of irAEs induced by immune checkpoint inhibitors used in the treatment of various cancers.
	Artacho A et al. [79]	2024	Ciprofloxacin, Meropenem, etc.	Boruta algorithm, RF:34 functional gene features34 functional gene features AUC = 0.88; Partial Least Squares-Correspondence Analysis	The study established a model to identify gut microbiota features associated with complications such as graft-versus-host disease and infections induced by allogeneic hematopoietic stem cell transplantation in the treatment of various hematological malignancies.
	Lötsch J et al. [80]	2024	Paclitaxel	PCA, Emergent Self-Organizing Map (ESOM), SVM, RF, Logistic Regression: The median balanced accuracy of the three algorithms reached up to 90%	The study found that sphinganine-1-phosphate may serve as a potential co-therapeutic target for alleviating chemotherapy-induced peripheral neuropathy caused by paclitaxel in the treatment of breast cancer and other types of cancers.
	Li ZC et al. [81]	2024	Lenvatinib and anti-PD1 monoclonal antibody	Logistic Regression: AUC = 1.000; RF: AUC = 0.944	Predicting the treatment response of hepatocellular carcinoma patients treated with Lenvatinib combined with anti-PD1 antibodies based on plasma metabolomic features.
	Moreno-Torres M et al. [82]	2024	Epistane, oxaliplatin, etc.	PLS-DA: The quantitative accuracy of residual abnormality detection reaches the “percentage-level”	The study focuses on DILI associated with 31 different drugs and has established PLS-DA models that can accurately classify DILI subtypes and quantify the contributions of these subtypes.
	Yu SM et al. [83]	2024		LASSO: The quantitative accuracy of residual abnormality detection reaches the “percentage-level”; RF: AUC = 0.969	A variety of machine learning models have been established for DILI diagnosis and marker screening.
	Chen CS et al. [84]	2024	Paclitaxel	LR, LinR, ORA, DSPCN; ML: poor predictive performance	Profiled 20 pretreatment serum amino acids in breast cancer patients; weak univariate associations with CIPN vanished after multivariate adjustment; no stable predictive metabolic biomarkers obtained
	Zhang Y et al. [85]	2025	Emodin	Double random forest (RF-RF): Training accuracy = 1.00, Test accuracy = 0.97, Precision = 0.99; PCA, PLS-DA, etc.	The research focuses on liver metabolic disorders induced by emodin (exploring its hepatotoxic mechanism).
	Su X et al. [86]	2025	methylprednisolone	RF: Classification accuracy, model constructed with 3 bacterial genera, AUC = 0.91	This study investigates the effects of high-dose glucocorticoid therapy on the gut microbiota and metabolome of patients with Graves’ ophthalmopathy, providing new insights into the microbiota-mediated glucocorticoid toxicity mechanisms.
	Liu Y et al. [87]	2025	Cyclophosphamide	PCA: VIP > 1, *p* < 0.05; OPLS-DA: VIP > 1, *p* < 0.05	Shenjiao Lingcao Decoction (a Chinese herbal compound) is used to improve cyclophosphamide-induced immunosuppression and spleen damage.
	Tian W et al. [88]	2026	Sintilimab	RF; XAI(SHAP) for feature interpretation, optimal AUC ≥ 0.80	Combining plasma metabolomics with machine learning predicts sintilimab-triggered rash in lung cancer patients, where SHAP (XAI tool) quantifies metabolite contribution to screen predictive biomarkers.

## Data Availability

No new data were created or analyzed in this study.
